# Risk to Human Health from a Plethora of Simian Immunodeficiency Viruses in Primate Bushmeat

**DOI:** 10.3201/eid0805.01-0522

**Published:** 2002-05

**Authors:** Martine Peeters, Valerie Courgnaud, Bernadette Abela, Philippe Auzel, Xavier Pourrut, Frederic Bibollet-Ruche, Severin Loul, Florian Liegeois, Cristelle Butel, Denis Koulagna, Eitel Mpoudi-Ngole, George M. Shaw, Beatrice H. Hahn, Eric Delaporte

**Affiliations:** * Institut de Recherche pour le Développement (IRD), Montpellier, France; † Projet Prevention du Sida au Cameroun (PRESICA), Yaounde, Cameroon; ‡ Faculté Universitaire des Sciences Agronomiques de Gembloux, Gembloux, Belgium; § University of Alabama at Birmingham, Birmingham, Alabama, USA; ¶ Ministry of Environment and Forestry, Yaounde, Cameroon

## Abstract

To assess human exposure to *Simian immunodeficiency virus* (SIV) in west central Africa, we looked for SIV infection in 788 monkeys that were hunted in the rainforests of Cameroon for bushmeat or kept as pets. Serologic reactivity suggesting SIV infection was found in 13 of 16 primate species, including 4 not previously known to harbor SIV. Overall, 131 sera (16.6%) reacted strongly and an additional 34 (4.3%) reacted weakly with HIV antigens. Molecular analysis identified five new phylogenetic SIV lineages. These data document for the first time that a substantial proportion of wild monkeys in Cameroon are SIV infected and that humans who hunt and handle bushmeat are exposed to a plethora of genetically highly divergent viruses.

First recognized in the early 1980s, AIDS represents the endstage of infection with one of two lentiviruses, termed *Human immunodeficiency virus* *type 1* (HIV-1) or *type 2* (HIV-2) (*1*,*2*). HIV-1 has spread to most parts of the world, while HIV-2 has remained largely restricted to West Africa (*3*,*4*). More than 40 million persons are estimated to have HIV infection or AIDS (*4*).

Both HIV-1 and HIV-2 are of zoonotic origin (*5*). The closest simian relatives of HIV-1 and HIV-2 have been found in the common chimpanzee (*Pan troglodytes)* and the sooty mangabey (*Cercocebus atys*), respectively (*6*–*8*), and phylogenetic evidence indicates that lentiviruses from these species (SIVcpz and SIVsm, respectively) have been transmitted to humans on at least eight occasions (*5*,*9*). Serologic evidence of SIV infection has so far been documented in 26 primate species, and 20 of these viruses have been at least partially molecularly characterized (*5*,*10*,*11*). Because humans come in frequent contact with primates in many parts of subSaharan Africa, additional zoonotic transfers of primate lentiviruses from species other than chimpanzees and sooty mangabeys are possible. The risk for acquiring SIV infection would be expected to be highest in persons who hunt primates and prepare their meat for consumption, as well as in persons who keep primates as pets. However, this risk cannot be assessed since the prevalence, diversity, and geographic distribution of SIV infections in wild primate populations are unknown. We report the first comprehensive survey of wild-caught primates in Cameroon, home to diverse primate species that are extensively hunted for food and trade (*12*). Much of the primate meat sold for consumption derives from infected monkeys, and a comparable number of pet monkeys also carry SIV. These data thus provide a first approximation of the magnitude and variety of SIVs to which humans are exposed through contact with nonhuman primates.

## Materials and Methods

### Collection of Primate Tissue and Blood Samples

Blood was obtained from 788 monkeys wild-caught in Cameroon from January 1999 to April 2001. Species were determined by visual inspection according to the Kingdon Field Guide to African Mammals (*13*) and the taxonomy described by Colin Groves (*14*). We sampled 573 animals as bushmeat at markets in Yaounde (n=157), surrounding villages (n=111), or logging concessions in southeastern Cameroon (n=305), as well as 215 pet animals from these same areas (Table 1). All primate samples were obtained with government approval from the Cameroonian Ministry of Environment and Forestry. Bushmeat samples were obtained through a strategy specifically designed not to increase demand: women preparing and preserving the meat for subsequent sale and hunters already involved in the trade were asked for permission to sample blood and tissues from carcasses, which were then returned.

**Table 1:** Wild-born primates surveyed, by species, age, and status, Cameroon

Genus	Species	Common name	Pet animals		Primate bushmeat		Total
			Adults	Juveniles/ Infants	Adults	Juveniles/ infants	
*Cercocebus*	*agilis*	Agile mangabey	4	15	30	3	52
	*torquatus*	Red-capped mangabey	1	–	–	1	2
*Lophocebus*	*albigena*	Grey-cheeked mangabey	3	3	12	3	21
*Cercopithecus*	*cephus*	Mustached guenon	3	26	217	56	302
	*mona*	Mona monkey	–	7	1	1	9
	*neglectus*	De Brazza’s monkey	2	6	21	5	34
	*nictitans*	Greater spot-nosed monkey	8	36	110	12	166
	*pogonias*	Crested mona	1	5	57	10	73
	*preussi*	Preuss’s monkey	–	1	–	–	1
*Chlorocebus*	*tantalus*	Tantalus monkey	7	11	–	–	18
*Miopithecus*	*ogouensis*	Gabon talapoin	5	6	8	–	19
*Erytrocebus*	*patas*	Patas monkey	5	14	–	–	19
*Colobus*	*guereza*	Mantled guereza	**–**	2	24	–	26
*Mandrillus*	*leucophaeus*	Drill	–	2	–	–	2
	*sphinx*	Mandrill	5	15	–	2	22
*Papio*	*anubis*	Olive baboon	11	11	–	–	22
Total			55	160	480	93	788

For the bushmeat animals, blood was collected by cardiac puncture, and lymph node and spleen tissues were collected whenever possible. The owners indicated that most of the animals had died 12 to 72 hours before sampling. For pet monkeys, blood was drawn by peripheral venipuncture after the animals were tranquilized with ketamine (10 mg/kg). Plasma and cells were separated on site by Ficoll gradient centrifugation. All samples, including peripheral blood mononuclear cells (PBMCs), plasma, whole blood, and other tissues, were stored at –20°C.

### Serologic Testing

Plasma samples were tested for HIV/SIV antibodies by the INNO-LIA HIV Confirmation test (Innogenetics, Ghent, Belgium), which includes HIV-1 and HIV-2 recombinant proteins and synthetic peptides that are coated as discrete lines on a nylon strip. Five HIV-1 antigens include synthetic peptides for the exterior envelope glycoprotein (sgp120), as well as recombinant proteins for the transmembrane envelope glycoprotein (gp41), integrase (p31), core (p24), and matrix (p17) proteins. HIV-1 group O envelope peptides are included in the HIV-1 sgp120 band. The HIV-2 antigens include synthetic peptides for sgp120, as well as recombinant gp36 protein. In addition to these HIV antigens, each strip has control lines: one sample addition line (3+) containing anti-human immunoglobulin (Ig) and two test performance lines (1+ and +/-) containing human IgG. All assays were performed according to manufacturer’s instructions, with alkaline phosphatase-labeled goat anti-human IgG as the secondary antibody. We used the following working definition for SIV seropositivity: plasma samples were scored as INNO-LIA positive when they reacted with at least one HIV antigen and had a band intensity equal to or greater than the assay cutoff (+/-) lane; samples that reacted less strongly but still visibly with two or more HIV antigens were classified as indeterminant; and samples reacting with no bands or only one band with less than +/- intensity were classified as negative.

### Polymerase Chain Reaction (PCR)

DNA was isolated from whole blood or PBMCs by using Qiagen DNA extraction kits (Qiagen, Courtaboeuf, France), and PCR was done with the Expand High Fidelity PCR kit (Roche Molecular Biochemicals, Mannheim, Germany). For amplification of SIV sequences, previously described degenerate consensus *pol* primers DR1, Polis4, UNIPOL2, and PolOR (*15*–*17*) were used in various combinations under previously described PCR conditions (*16*). PCR products were sequenced by cycle sequencing and dye terminator methods (ABI PRISM Big Dye Terminator Cycle Sequencing Ready Reaction kit with AmpliTaq FS DNA polymerase [PE Biosystems, Warrington, England]) on an automated sequencer (ABI 373, Stretch model; Applied Biosystems, Courtaboeuf, France) either directly or after cloning into the pGEM-T vector (Promega, Charbonnieres, France).

To test for DNA degradation, a 1,151-bp region of the glucose-6–phosphate dehydrogenase (G6PD) gene was amplified with the primers GPD-F1 5′-CATTACCAGCTCCATGACCAGGAC-3′ and GPD-R1 5′-GTGTTCCCAGGTGACCCTCTGGC-3′ in a single-round PCR reaction under the following conditions: 94°C for 2 min, then 35 cycles at 94°C for 20 sec; 58°C for 30 sec, and 72 °C for 1 min (*18*).

### Phylogenetic Analyses

Newly derived SIV nucleotide sequences were aligned with reference sequences from the Los Alamos HIV/SIV Sequence database by using CLUSTAL W (*19*) with minor adjustments for protein sequences. A phylogenetic tree was constructed by the neighbor-joining method (*20*), and the reliability of branching orders was tested by the bootstrap approach (*21*). Sequence distances were calculated by Kimura’s two-parameter method (*22*). SIV lineages were defined as clusters of SIV sequences from the same primate species that grouped together with significant (>80%) bootstrap values.

### GenBank Accession Numbers

The new sequences have been deposited in GenBank under the following accession numbers: SIVgsn-99CM-CN71 (AF478588), SIVgsn-99CM-CN7 (AF478589), SIVgsn-99CM-CN166 (AF478590), SIVmon-99CM-CML1 (AF478591), SIVmus-01CM-S1239 (AF478592), SIVmus-01CM-S1085 (AF478593), SIVtal-00CM-271 (AF478594), SIVtal-00CM-266 (AF478595), SIVmnd2-99CM-54 (AF478596), SIVmnd2-01CM-S109 (AF478597), SIVmnd2-00CM-S46 (AF478598), SIVmnd2-00CM-S6 (AF478599), SIVdeb-01CM-1083 (AF478600), SIVdeb-99CM-CN40 (AF478601), SIVdeb-01CM-S1014 (AF478602), SIVdeb-99CM-CNE5 (AF478603), SIVdeb-01CM-1161 (AF478604), SIVdeb-99CM-CNE1 (AF478605), SIVcol-00CM-247 (AF478606), SIVcol-00CM-243 (AF478607), and SIVcol-99CM-11 (AF478608).

## Results

### Prevalence Estimates of SIV Infection in Bushmeat and Pet Monkey Samples

Previous studies of SIV infection have relied almost exclusively on surveys of captive monkeys or apes that were either kept as pets or housed at zoos, sanctuaries, or primate centers. While this approach has led to the discovery of novel SIVs (*23*–*29*), it has not provided information concerning SIV prevalence rates in the wild. Most pet monkeys are acquired at a very young age, often when their parents are killed by hunters. Two field studies of wild African green monkeys have shown that seroprevalence rates correlated with sexual maturity, suggesting transmission predominantly by sexual routes (*30*,*31*). SIV infection rates of captive monkeys may thus not accurately reflect SIV prevalence rates in the wild.

**Figure 1:**
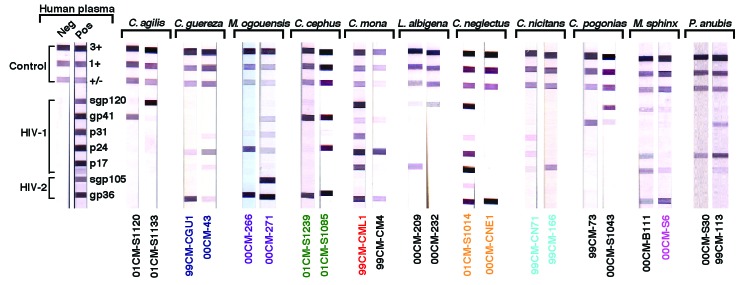
Detection of HIV-1/HIV-2 cross-reactive antibodies in sera from 11 primate species by using a line immunoassay (INNO-LIA HIV Confirmation, Innogenetics, Ghent, Belgium). Varying patterns of reactivity to HIV peptides and proteins (HIV-1 gp120, gp41, p31, p24, and p17; HIV-2 gp130, and gp36) are shown. Samples from which *Simian immunodeficiency virus* (SIV) sequences were subsequently amplified by polymerase chain reaction are color-coded as in Figure 2. Plasma samples from HIV-1/HIV-2-negative and -positive persons are shown as controls on the left. The 3+, 1+ and +/- bands at the top of all test strips control for sample addition (presence of plasma immunoglobulin) and test performance (binding of secondary antibody).

To ensure systematic sampling, we therefore collected blood from 573 monkeys sold as bushmeat and 215 pet monkeys (Table 1). Most of the bushmeat animals were adults, while most of the pets were still infants or juveniles at the time of sampling. Most primates came from the southern part of the country. All major SIV lineages known to date were initially discovered because their primate hosts had antibodies that cross-reacted with HIV-1 or HIV-2 antigens (*23*–*29*). Although the extent of this cross-reactivity has not been defined, we used a similar approach to examine the primate blood samples obtained in Cameroon. Since commercially available HIV screening assays (e.g., enzyme-linked immunosorbent assay or rapid tests) contain only a limited number of antigens, we used an HIV confirmatory assay (INNO-LIA), comprising a recombinant and synthetic peptide-based line immunoassay (Figure 1). One hundred thirty-one (16.6%) of 788 plasma samples reacted strongly with one or more HIV antigens, while an additional 34 samples (4.3%) reacted less strongly but visibly with two or more HIV antigens (Figure 1; Table 2). Of 13 primate species that had HIV cross-reactive antibodies, the prevalence of seroreactivity (positive plus indeterminant) ranged from 5% to 40%. Prevalences were lower in pet animals than in bushmeat primates, 11.6% versus 18.4%, respectively. Sera from only three species failed to react completely (*Cercopithecus preussi*, *Mandrillus leucophaeus, Cercocebus torquatus*), but these three species accounted for only 5 of the 788 samples tested.

**Table 2:** HIV-1/HIV-2 cross-reactive antibodies^a^ detected in primate species hunted in Cameroon

Genus	Species	Common name	Pet animals		Primate bushmeat		Total	
			pos/tested	ind/tested	pos/tested	ind/tested	pos/tested	ind/tested
*Cercocebus*	*agilis*	Agile mangabey	1/19	1/19	5/33	7/33	6/52	8/52
	*torquatus*	Red-capped mangabey	0/1	0/1	0/1	0/1	0/2	0/2
*Lophocebus*	*albigena*	Grey-cheeked mangabey	0/6	0/6	2/15	3/15	2/21	3/21
*Cercopithecus*	*cephus*	Mustached guenon	1/29	3/29	48/273	9/273	49/302	12/302
	*mona*	Mona monkey	1/7	0/7	1/2	0/2	2/9	0/9
	*neglectus*	De Brazza’s monkey	1/8	0/8	9/26	1/26	10/34	1/34
	*nictitans*	Greater spot-nosed monkey	6/44	0/44	22/122	3/122	28/166	3/166
	*pogonias*	Crested mona	0/6	0/6	9/67	4/67	9/73	4/73
	*preussi*	Preuss’s monkey	0/1	-	-	-	0/1	-
*Chlorocebus*	*tantalus*	Tantalus monkey	3/18	0/18	-	-	3/18	0/18
*Miopithecus*	*ogouensis*	Gabon talapoin	2/11	1/11	2/8	0/8	4/19	1/19
*Erythrocebus*	*patas*	Patas monkey	1/19	0/19	-	-	1/19	0/19
*Colobus*	*guereza*	Mantled guereza	0/2	0/2	7/24	1/24	7/26	1/26
*Mandrillus*	*leucophaeus*	Drill	0/2	0/2	-	-	0/2	0/2
	*sphinx*	Mandrill	7/20	0/20	1/2	1/2	8/22	1/22
*Papio*	*anubis*	Olive baboon	2/22	0/22	-	-	2/22	0/22
Total			25/215	5/215	106/573	29/573	131/788	34/788
(%)			11.6%	2.3%	18.4%	5.1%	16.6%	4.3%

**^a^**Plasma samples were tested for antibodies cross-reactive with HIV-1 and HIV-2 antigens by using a recombinant-based line immunoassay (INNO-LIA HIV Confirmation, Innogenetics, Ghent, Belgium). Positive (pos) and indeterminant (ind) INNO-LIA scoring criteria as described in Methods.

The INNO-LIA profiles from members of the same as well as different primate species varied extensively (Figure 1). Some sera reacted only with HIV core and/or Pol proteins, while others reacted with Gag and/or Pol and/or Env proteins from either HIV-1 or HIV-2 or both. Other than classifying sera as INNO-LIA reactive or nonreactive, no banding pattern or algorithm could be derived that would have been predictive of infection of any given primate species.

### Confirmation of SIV Infection by PCR and Discovery of Novel SIV Lineages

**Figure 2:**
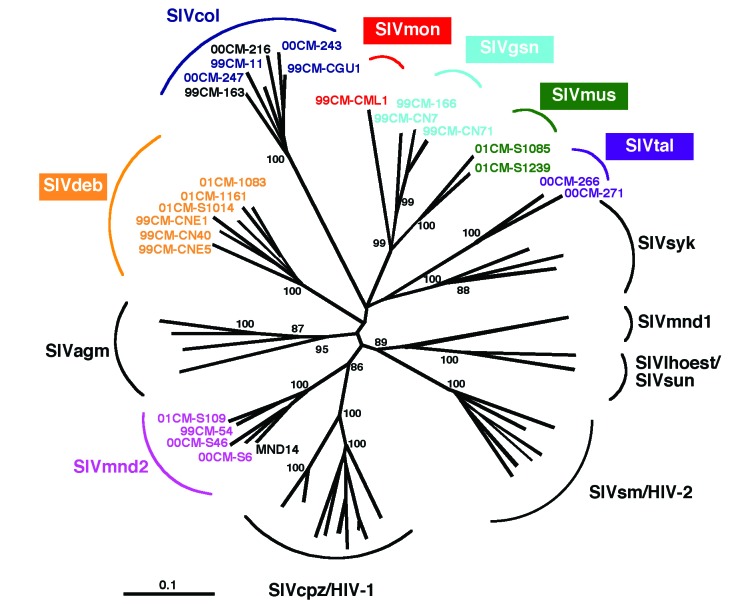
Identification of diverse *Simian immunodeficiency virus* (SIV) lineages in primate bushmeat. A 650-bp *pol* fragment was amplified from monkeys representing seven primate species, sequenced, and subjected to phylogenetic tree analysis by the neighbor-joining method. The positions of 21 SIV sequences from the present study (in color) are shown in relation to HIV/SIV reference sequences from the Los Alamos HIV/SIV Sequence Database (in black). The consensus length of the final alignment used for tree construction was 555 bp. The new species-specific SIV lineages are generally identified by a lower-case three-letter code corresponding to the initial letters of the common species name (e.g., SIVgsn for greater spot-nosed monkeys [*Cercopithecus nictitans*], SIVmus for mustached guenons [*C. cephus*] and SIVmon for mona monkeys [*C. mona*]). Lineages are defined as clusters of viral sequences from the same primate species that group together with significant (>80%) bootstrap values. We maintained the lineage designation of SIVtal previously assigned to a virus thought to be derived from a zoo animal of the species *Miopithecus talapoin* (*28*) since that sequence, and the two newly derived talapoin viruses from *M. ogouensis,* cluster together in a phylogenetic tree derived from additional *pol* nucleotide sequences (not shown). Branch lengths are drawn to scale (the bar indicates 10% divergence). The numbers at the nodes indicate the percent bootstrap values supporting the cluster to the right (only values >80% are shown).

A total of 342 samples, including INNO-LIA positive (n=91), indeterminant (n=23), or negative (n=228) specimens were subjected to PCR analysis (16,32), which yielded amplification products for 28 blood samples from seven primate species: *Cercopithecus mona*, *C. neglectus*, *C. nictitans*, *C. cephus, Colobus guereza, Miopithecus ogouensis,* and *Mandrillus sphinx* (Table 3). All these amplification products were of appropriate size. Moreover, subsequent sequence and phylogenetic analysis confirmed SIV infection (Figure 2). Most of the newly derived sequences did not fall into any of the known SIV groups. Viral sequences from *C. mona* (SIVmon), *C. neglectus* (SIVdeb), *C. nictitans* (SIVgsn), *C. cephus* (SIVmus), and *Miopithecus ogouensis* (SIVtal) formed species-specific monophyletic clusters that were roughly equidistant from each other as well as from all previously defined SIV lineages in this region of the *pol* gene. Viruses from the remaining two species (*Colobus guereza* and *Mandrillus sphinx*) grouped with previously reported SIVcol and SIVmnd-2 strains, respectively.

**Table 3:** Polymerase chain reaction (PCR) amplification of *Simian immunodeficiency virus* (SIV) sequences

Genus	Species	INNO-LIA pos^a^ PCR pos/tested	INNO-LIA ind PCR pos/tested	INNO-LIA neg PCR pos/tested
*Cercocebus*	*agilis*	0/6	0/8	0/13
	*torquatus*	–	–	0/1
*Lophocebus*	*albigena*	0/2	0/2	0/7
*Cercopithecus*	*cephus*	2/25	0/7	0/56
	*mona*	½	–	0/2
	*neglectus*	8/9	–	0/4
	*nictitans*	3/21	1/1	0/61
	*pogonias*	0/9	0/3	0/34
*Chlorocebus*	*tantalus*	0/1	–	0/2
*Miopithecus*	*ogouensis*	2/3	–	0/10
*Erythrocebus*	*patas*	–	–	0/7
*Colobus*	*guereza*	6/6	0/1	1/16
*Mandrillus*	*sphinx*	4/5	0/1	0/4
*Papio*	*anubis*	0/2	–	0/11
Total		26/91	1/23	1/228

^a^DNA was extracted from a subset of seropositive (pos), indeterminant (ind) and negative (neg) blood samples and subjected to nested PCR amplification by using HIV/SIV consensus *pol* primer pairs. In each column, the number of PCR-positive samples per total number of samples tested is indicated. The authenticity of all amplification products was confirmed by sequence analysis.

The single sequence of SIVmon was given lineage status because of its high degree of genetic diversity from the other SIV strains. We maintained the lineage designation of SIVtal previously assigned to a virus thought to be derived from a zoo animal of the species *M. talapoin* (*28*) because that sequence and the two newly derived talapoin viruses from *M. ogouensis* cluster together in a phylogenetic tree derived from additional *pol* nucleotide sequences (not shown). Thus, our new SIVtal sequences confirm the existence of this lineage in the wild .

SIV sequences were confirmed in 26 of 91 INNO-LIA-positive samples, as well as in 1 of 23 indeterminate and 1 of 223 negative samples (Table 3). Because many blood samples were obtained under poorly controlled circumstances, especially from the bushmeat markets, we tested the possibility of DNA degradation. Whole blood and PBMC DNA preparations were subjected to single-round PCR with primers designed to amplify introns 4 and 5 of the nuclear G6PD gene (1,100 bp). Of the 65 LIA-positive samples that did not yield a virus-specific PCR product, 11 also failed to yield a G6PD amplification product. Similarly, 4 of 17 INNO-LIA-indeterminate and SIV PCR-negative samples, as well as 25 of 102 INNO-LIA-negative samples, were also negative by G6PD amplification. These results indicate that, in addition to using only a single set of nested *pol* primer pairs, low PCR amplification rates from LIA-positive and -indeterminant samples were also due to DNA degradation, the presence of PCR inhibitors, or both.

## Discussion

Zoonotic transfers of SIV to humans have been documented on no fewer than eight occasions (*5*,*9*), but no previous study has examined to what extent African primates that are frequently hunted or kept as pets are infected with SIV. Although our serologic screening approach has limitations (i.e., an unknown extent of antigenic cross-reactivity between HIV proteins and SIV antibodies), we were able to detect cross-reactive antibodies suggesting SIV infection in 16.6% of all tested animals, including members of four species not previously known to harbor SIV (*C. agilis*, *Lophocebus albigena*, *C. pogonias,* and *Papio anubis*). PCR confirmation and molecular identification of SIV infection were obtained in seven species, and phylogenetic analyses showed the presence of highly divergent viruses that grouped according to their species of origin. Four of these SIV lineages from mona *(C. mona*), De Brazza’s (*C. neglectus*), mustached (*C.* *cephus*), and greater spot-nosed (*C. nictitans*) monkeys have not previously been recognized. Finally, we confirmed the SIVtal infection of wild talapoin monkeys (*Miopithecus ogouensis*). These data establish for the first time that a considerable proportion of wild-living primates in Cameroon are infected with SIV, posing a potential source of infection to those who come in contact with them. Our findings bring to 30 the number of African nonhuman primate species known or strongly suspected to harbor primate lentiviruses (*5*)*.*

Our data likely still underestimate the prevalence and diversity of naturally occurring SIV infections in Cameroon. First, not all native primate species were tested, and many were undersampled because they were either rare in the regions of Cameroon where we sampled for this study or too small to be regularly hunted. For example, the absence of reactive sera from drills and red-capped mangabeys, two species known to harbor SIV (*15*,*23*), must be due to the low number of blood samples (5/788) analyzed. In addition, the INNO-LIA test sensitivity is clearly not 100%, as one negative sample contained SIV sequences as determined by PCR amplification. Finally, our PCR approach, which utilized only a single set of nested primers, likely amplified only a subset of viral sequences. Thus, the true prevalence of SIV infection in the various primate species will require the development of SIV lineage-specific assays with known sensitivities and specificities.

Human infection with SIVcpz and SIVsm is thought to have resulted from cutaneous or mucous membrane exposure to infected blood during the hunting and butchering of chimpanzees and sooty mangabeys for food (*5*). Bites from pet animals and possibly contact with fecal and urine samples may have also been involved (*5*). Our study shows that many primate species in addition to chimpanzees and sooty mangabeys are hunted and that 20% (or more) of these animals likely harbor SIV. Thus, if contact with infected blood or other secretions is indeed the primary route of transmission, hunters and food handlers may be at risk of infection with many more SIVs than just those from chimpanzees and sooty mangabeys.

Bushmeat hunting, to provide animal proteins for the family and as a source of income, has been a longstanding common component of household economies in the Congo Basin and, more generally, throughout subSaharan Africa (*33*–*35*). However, the bushmeat trade has increased in the last decades. Commercial logging, which represents an important economic activity in Cameroon as well as many other west-central African countries, has led to road constructions into remote forest areas, human migration, and social and economic networks supporting this industry (*36*). Hunters are now penetrating previously inaccessible forest areas, making use of newly developed infrastructure to capture and transport bushmeat from remote areas to major city markets (*37*). Moreover, villages around logging concessions have grown from a few hundred to several thousand inhabitants in just a few years (*37*). These socioeconomic changes, combined with our estimates of SIV prevalence and genetic complexity in wild primates, suggest that the magnitude of human exposure to SIV has increased, as have the social and environmental conditions that would be expected to support the emergence of new zoonotic infections.

Whether any of the newly identified SIVs have the ability to infect humans remains unknown since molecular evidence is lacking for SIV cross-species transmissions from primates other than chimpanzees and sooty mangabeys. However, such infections may have been unrecognized by HIV-1/HIV-2 screening assays. A case in point is the recent identification of a Cameroonian man who had an indeterminant HIV serology but reacted strongly (and exclusively) with an SIVmnd V3 loop peptide (*32*). Although viral sequences were not confirmed in this man, the finding suggests that at least some naturally occurring SIVs have the potential to cross the species into the human population. In fact, several recently reported SIV isolates, including SIVlhoest, SIVsun, SIVrcm, and SIVmnd2, replicate well in primary human lymphocytes in vitro (*23*,*26*,*27*,*32*,*38*) as do SIVcpz (*25*) and SIVsm (*24*). Thus, to determine whether additional zoonotic transmissions of SIVs have already occurred, virus type- and/or lineage-specific immunoassays and PCRs will have to be developed. Such work should receive high priority given the extent of human exposure to different SIV lineages as a result of the expanding bushmeat trade and the impact of two major human zoonoses (HIV-1 and HIV-2). Recombination between newly introduced SIVs and circulating HIVs poses still another human risk for novel zoonoses.

In summary, the current HIV-1 pandemic provides compelling evidence for the rapidity, stealth, and clinical impact that can be associated with even a single primate lentiviral zoonotic transmission event. We document for the first time that humans are exposed to a plethora of primate lentiviruses through hunting and handling of bushmeat in Cameroon, a country at the center of HIV-1 groups M, N, and O endemicity that is home to a diverse set of SIV-infected nonhuman primates. To what extent wild monkey populations in other parts of Africa are also infected with diverse SIVs is unknown. A complete and accurate assessment of all SIV-infected nonhuman primate species is needed, as well as a determination of the virus lineage(s) present in each species. Studies are also needed to determine whether zoonotic transmissions of SIVs from primates other than chimpanzees and mangabeys have already occurred and what clinical outcomes were associated with these infections. Results from these studies will yield critical insights into the circumstances and factors that govern SIV cross-species transmission and thus allow determination of human zoonotic risk for acquiring these viruses.
